# Flame-Assisted Laser Polishing of Alumina Ceramic Surface Properties

**DOI:** 10.3390/mi14030520

**Published:** 2023-02-23

**Authors:** Ting Guo, Chao Wang, Junyong Zeng, Wenqi Wang, Zhenyu Zhao

**Affiliations:** 1 School of Sino-German Robotics, Shenzhen Institute of Information Technology, Shenzhen 518029, China; 2 School of Mechanical Engineering, Xiangtan University, Xiangtan 411105, China; 3 College of Mechatronics and Control Engineering, Shenzhen University, Shenzhen 518060, China

**Keywords:** response surface method, laser polishing, alumina ceramics, roughness, preheating

## Abstract

Laser polishing was used to reduce the surface roughness and improve the surface properties of alumina ceramics. In this paper, a response surface experimental design scheme is used to establish a mathematical model based on the Box–Behnken central combination principle, with the surface roughness as the optimization target to optimize the optimal process parameters for the laser polishing of alumina ceramics, to suppress the polished surface cracks by preheating the material, and to study the changes of surface properties of laser-polished alumina ceramics under different preheating temperatures. The optimal laser polishing process parameters were optimized by response surface experiments with a scanning speed of 323.5 mm/s, a laser power of 73.63 W, a pulse frequency of 2.3 kHz, and a scanning spacing of 0.09 mm; compared with the initial surface roughness of 4.67 μm, the polished surface roughness was 0.96 μm under the experimentally optimized polishing parameters, and the surface cracks were suppressed after the preheating treatment. The surface roughness was further reduced to 0.74 μm, and the surface wear coefficient was reduced from 0.5939 to 0.5725, while the surface hardness was increased from 1810 to 2063 HV. Optimization of the laser polishing process parameters through the response surface can significantly reduce the surface roughness of the material, while the flame preheating, assisted by the laser-polished surface wear resistance and hardness, is improved.

## 1. Introduction

Alumina ceramics are widely used in aviation, aerospace, and biomedical fields by virtue of their excellent properties, such as high melting point, high hardness, wear resistance, and corrosion resistance [1]. However, the traditional thermal sintering process produces alumina ceramic parts with a rough surface that cannot be directly applied, while the high hardness and good wear resistance make it difficult and inefficient to use conventional processing. This limits the use of alumina ceramic materials in the industry. With the development of technology, new processing methods such as ultrasonic polishing and laser polishing, which overcome the difficulties of traditional processing methods for high-precision processing, effectively improve the quality of the processed surface. Zhang et al. [1,2,3] used ultrasonic waves to polish the material surface; compared with traditional polishing methods, the use of ultrasonic polishing can effectively reduce the surface roughness of the material. However, due to the complexity of the technology and the material removal mechanism, which is not yet completely clear, it is not possible to optimize the process parameters effectively, which limits its widespread use. Laser polishing is a non-contact polishing method with a fast polishing speed compared to the traditional polishing process; it has a wide range of polishing objects and the ability to polish complex workpiece surfaces, and it emits no pollution into the environment.

At present, the laser polishing mechanism can be mainly divided into two types. The first mechanism is based on the photothermal mechanism—through the long pulse laser beam that causes the material surface heating, the material absorbs energy; the surface material redistributes under the action of gravity, surface tension, etc.; the surface material flows from the contour of the high place to the low place, and the material surface gradually smoothens. The second mechanism is based on the photochemical mechanism, mainly through an ultrafast laser on the material surface radiation; the pulse width is much smaller than the thermal diffusion time and the electron-phonon coupling time in the material, so the energy generated by the laser-absorbing photons when the pulse laser is operating is rapidly accumulated in a layer only a few nanometers thick, and the temperature of the instantly generated electrons far exceeds the material melting and evaporation temperature, which makes the material directly transform from a solid to a gaseous state to achieve the removal of the surface material, thus achieving the surface polishing effect [4].

In recent years, a large number of scholars at home and abroad have conducted experimental studies on the laser polishing of different materials, including metals and glass [5,6,7,8,9]. However, due to the high melting point and the low creep rate of the ceramic materials themselves, there are fewer reports on the use of laser polishing in ceramics. Bharatish et al. [10] used a CO_2_ laser for the surface treatment of 92% alumina ceramics and investigated the effect of laser process parameters. The results showed that the lowest indicated roughness of 0.60 μm could be obtained using a laser power of 90 W, a pulse frequency of 5 kHz, and a scanning speed of 333.37 mm/s. Tsai et al. [11,12,13] produced a smooth polished surface by removing the surface of Al_2_O_3_ ceramics through laser thermal stress. Ihlemann et al. [14] studied the ablation of different oxide ceramics using a UV laser with different pulse width lasers, and the results showed that the material is mainly dominated by thermally induced ablation when ceramics are processed using ns laser pulses; processing with an fs laser is dominated by multiphoton absorption. Umer et al. [15] used an Nd-YAG laser for a micro-milling study on alumina ceramic surfaces; the results showed that the laser pulse intensity and pulse overlap rate have a significant effect on surface roughness, while the surface material removal rate is mainly influenced by the laser beam intensity. The above studies show that the laser polishing of ceramic materials can significantly reduce the surface roughness of ceramics; however, the laser polishing process of ceramics often leads to cracks due to thermal stress, which reduces the surface properties after polishing. Zhang et al. [16,17] used a picosecond laser to polish ceramic surfaces, and the polished surfaces were smooth and free of obvious defects, such as cracks.

Das et al. [18] investigated the effect of cooling rate on the crack density of laser-processed ceramic coatings and showed that the crack density of laser-treated surface ceramic coatings was significantly reduced when the material was preheated prior to laser remelting. Despite the inherent advantages of using ultrafast lasers to polish ceramic materials, ultrafast lasers are currently expensive and not suitable for large-scale use, so reducing or eliminating surface cracks on conventional-pulsed laser-polished ceramic materials and improving polished surface properties are imperative. In this paper, the laser polishing of 99 alumina ceramics with a CO_2_ pulsed laser at a wavelength of 10.6 μm is studied, and the optimal laser polishing process parameters are optimized by the response surface method; the surface cracks are suppressed by the preheating method, and the surface properties of laser-polished alumina ceramics at different preheating temperatures are investigated.

## 2. Response Surface Methodology

### 2.1. Material Properties

The material used in the experiments was the 99-alumina ceramic (manufacturer: Shenzhen Hyde Ceramics Precision Co., Ltd. Shenzhen, China) produced by a sintering process; the specific chemical composition is shown in Table 1. The sample was 146 × 95 × 5 mm, and the experimental polishing was performed before the sample was put into the ultrasonic cleaner for 20 min.

### 2.2. Experimental Equipment

**Figure 1:**
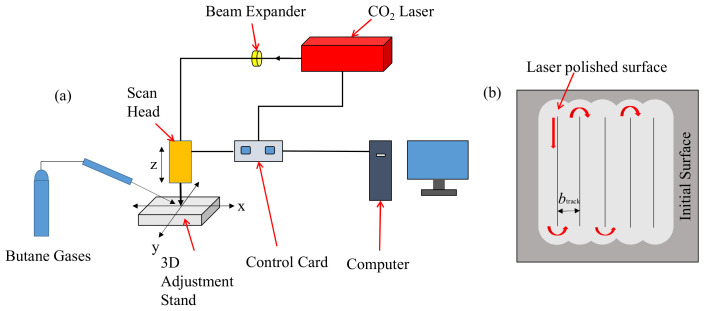
(**a**) Schematic diagram of laser polishing system. (**b**) Schematic diagram of scanning path.

The laser polishing system and scanning path used in the experiment are shown in Figure 1. The polishing system was mainly composed of a laser, a beam expander, a dynamic focusing oscillator, a three-dimensional adjustment frame, and a control computer, where the laser was a CO_2_ pulse laser (SYNRAD, FSTI100SWC, Seattle, DC, USA) with a wavelength of 10.6 μm, a laser output power of 0–150 W, a pulse width of 150–300 μs, and a frequency of 0–100 kHz. The dynamic focusing oscillator (Jinhaitron, RF8330-3D-1200, Jiangsu, China) had a focal length of 550 mm, a focusing spot of 0.314 mm, and a processable range of 400 × 400 mm. A laser confocal microscope (Mahr, MarSurf CM mobile, Göttingen, Germany) was used to examine the surface morphology of the material before and after polishing and the surface morphology of the friction wear track. A metallographic microscope (Siontae, CX200E, Shenzhen, China) and a field emission scanning electron microscope (ZEISS, Gemini 300, Gina, Germany) were used to observe the surface morphology before and after polishing. The material was heated directly by butane gas, and the preheating temperature was monitored in real-time by a portable temperature measuring gun (Sigma, AS872A, Dongguan, China). The abrasion resistance of the surface before and after polishing was tested by a friction and wear tester (Krundt, GF-1, Gansu, China). A residual stress tester (Proto, LXRD, Lachine, QC, Canada) was used to test the residual stress of the polished surface at different preheating temperatures, and an X-ray diffractometer (Bruker, D8 Advance, Billerica, Germany) was used to test the composition of the material phase before and after polishing.

### 2.3. Experimental Method

Before the polishing experiments, the original surface roughness of alumina ceramics was measured by laser confocal microscope; the test area was 5 × 5 mm, and the original surface roughness was 4.67 μm with a standard deviation of 0.5712. Response surface methodology is usually based on mathematical and experimental data to analyze multivariate optimization problems through statistics and seek the best combination of variables. This experiment aims to optimize the best process parameters of laser polishing and the effect of the interaction of factors on surface roughness by the response surface method. The polishing experiment adopts four factors and three levels of the experimental scheme. Table 2 shows the response surface experimental factor levels through the Design-Expert 8.0 software’s built-in response surface module for experimental design; a total of 27 sets of experiments was needed. Table 3 is the response surface experimental design and surface roughness; as can be seen directly from the table, the maximum value of surface roughness after laser polishing is 3.02 μm, and the minimum surface roughness value is 1.004 μm, which is 78% lower, relative to the original surface roughness.

## 3. Results and Analysis

### 3.1. Experimental Results of Response Surface Optimization

According to the experimental data in Table 3, the regression model equation of surface roughness is obtained by using the software’s experimental results for analysis through the surface roughness as the response index:

(1) $$\begin{array}{l} Ra=1.11-0.096v-0.092P+0.21f-0.31b\\ -0.21vP-0.082vf+0.46vb-0.32Pf-0.39Pb-0.45fb\\ +0.2v^{2}+0.58P^{2}+0.27f^{2}+0.66b^{2} \end{array}$$

**Figure 2:**
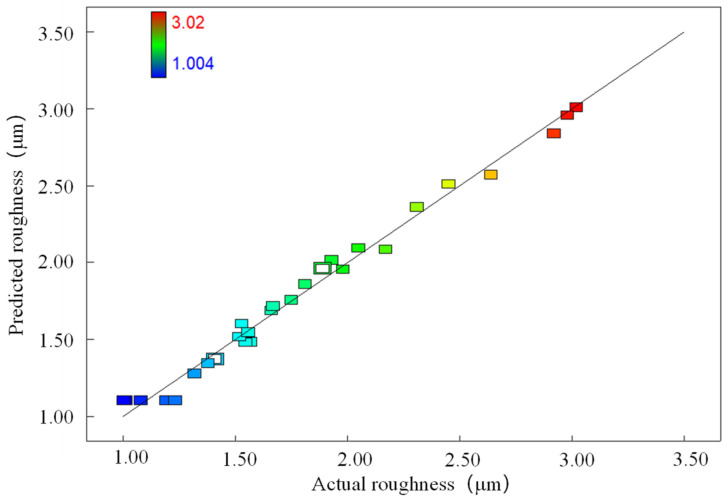
Predicted and actual values of surface roughness.

Table 4 shows the specific analysis of the variance of the experimental results using the software. From the table, it can be seen that for the laser frequency, the scanning spacing *P*_valve_ was <0.0001, while for the laser power, the scanning speeds *P*_valve_ were 0.0036, 0.0026 less than 0.05. Through the size of the *P*_valve_ value, it can be concluded that the laser polishing alumina ceramics used in the frequency and the scanning spacing on the material surface roughness are extremely significant. The effects of the laser power and scanning speed on the material surface roughness are more obvious, and the secondary terms are all more significant; in addition, the interaction term AB is significant, AC is not significant, and the rest of the interaction terms are more significant. In addition, the model *P*_valve_ < 0.0001 indicates that the response surface model reaches a highly significant level, and the loss of fit term *P*_valve_ is, at 0.7263, not significant, indicating that other factors have little effect on the surface roughness; the experimental results have good stability. Figure 2 gives a comparison fitting graph of the actual value of the experimental results and the predicted value of the model. The colored rectangle in the graph is the data point, its horizontal coordinate is the actual data, and the corresponding vertical coordinate is the predicted data. It can be seen from the graph that the predicted value of the model is very close to the results obtained from the experiment; the polishing model mutual coefficient R^2^ is 0.9873, and this parameter shows that the experimental model and the actual effect of polishing is a good fit. The fitting model can explain 98.73% of the actual polishing results, and the response surface polishing experimental model decision coefficient R^2^_Adj_ is 0.9746, which indicates that the response surface model has high accuracy and generality. It can be seen through the above parameters of the model that the established response surface model can accurately analyze and predict the relationship between the laser process parameters on the surface roughness after polishing.

**Figure 3:**
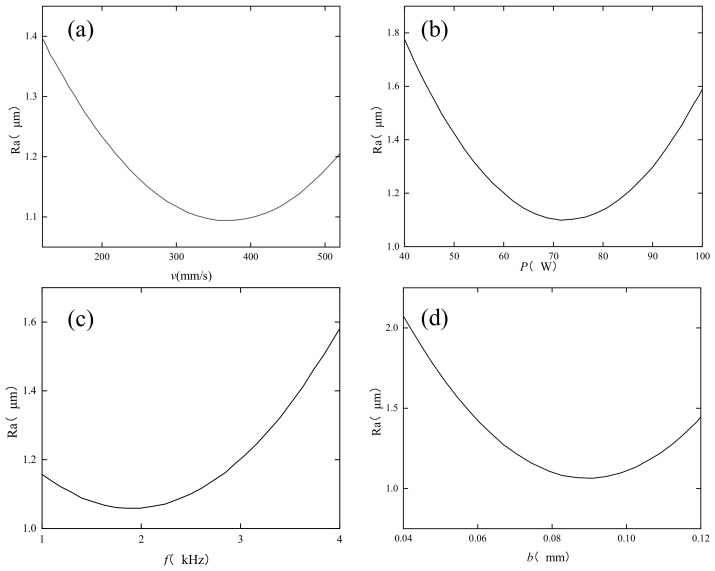
Effect of a single factor on surface roughness. (**a**) effect of scanning speed on surface roughness, (**b**) effect of laser power on surface roughness, (**c**) effect of pulse frequency on surface roughness, (**d**) effect of scanning spacing on surface roughness.

Figure 3 shows the effect of a single factor on surface roughness. From Figure 3a it can be seen that the surface roughness value, with the increase in scanning speed, first decreases and then increases in trend, mainly because the scanning speed directly affects the laser radiation surface time. If the speed is too fast, the surface material will be too short by the laser radiation time, the surface melt pool depth will be too shallow, and the smoothing effect will not be obvious. A scanning speed that is too slow will lead to a melt pool. If the temperature is too high, the surface roughness increases; combined with Figure 3b, it can be seen that the surface roughness, with an increase in laser power, shows a trend of first decreasing and then increasing, mainly because in the case of other parameters, the control is unchanged; increasing the laser power will make the laser flux increase; if the laser power is low, the surface material will not fully melt; the remelting leading to surface roughness reduction is not obvious; when continuing to increase the laser power, the surface roughness gradually decreases. However, when the power continues to increase, the excessive laser flux will lead to high surface temperatures and material evaporation, leading to a gradual increase in surface roughness. The pulse frequency in Figure 3c also decreases first and then increases, mainly because the pulse frequency mainly determines the size of the laser flux. Increasing the laser pulse frequency will make the single pulse laser flux decrease; the corresponding melt pool depth decreases on the material surface. The surface roughness increases due to insufficient melting. From Figure 3d, it can be seen that with the increase in the scan pitch, the surface roughness decreases and then increases; the scan pitch directly affects the overlap rate in the direction of the polishing trajectory. The scan pitch is too small, the overlap rate is too high, and the surface material is processed several times, resulting in increased thermal effects; the material surface roughness increases.

**Figure 4:**
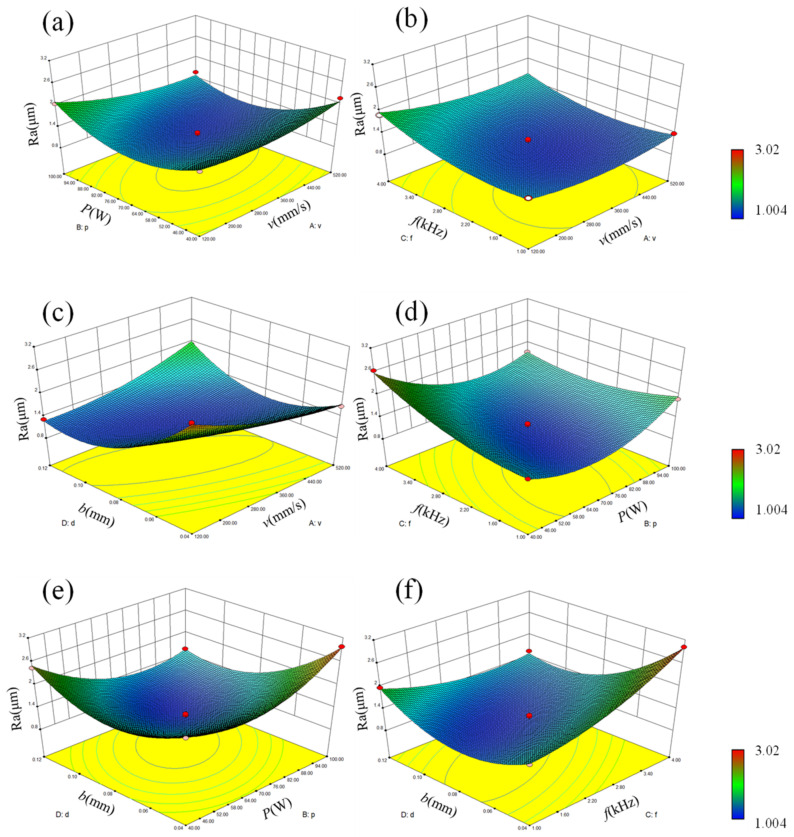
Effect of laser polishing interaction process parameters on surface roughness. (**a**) effect of AB interaction on surface roughness, (**b**) effect of AC interaction on surface roughness, (**c**) effect of AD interaction on surface roughness, (**d**) effect of BC interaction on surface roughness, (**e**) effect of BD interaction on surface roughness, and (**f**) effect of CD interaction on surface roughness.

In order to investigate the effect of laser polishing process parameter interaction on surface roughness, the above quadratic regression equation and experimental ANOVA data were used to draw response surface 3D plots to analyze the effect of laser polishing process parameters on surface roughness. When two of the process parameters are at the center level, the other two process parameters interact on the surface roughness. Figure 4 represents the effect of interaction terms on the polished surface roughness. Theoretically, the steeper the response surface curve, the smaller the interaction term *P*_valve_, indicating that the interaction term has a greater effect on the response value. Combined with the interaction term *P*_valve_ in Figure 4 and Table 4, it can be seen that AD, BC, BD, and CD interactions have a large effect on surface roughness, while the AB interaction has the second largest effect on surface roughness and the AC interaction has the smallest effect on surface roughness.

**Figure 5:**
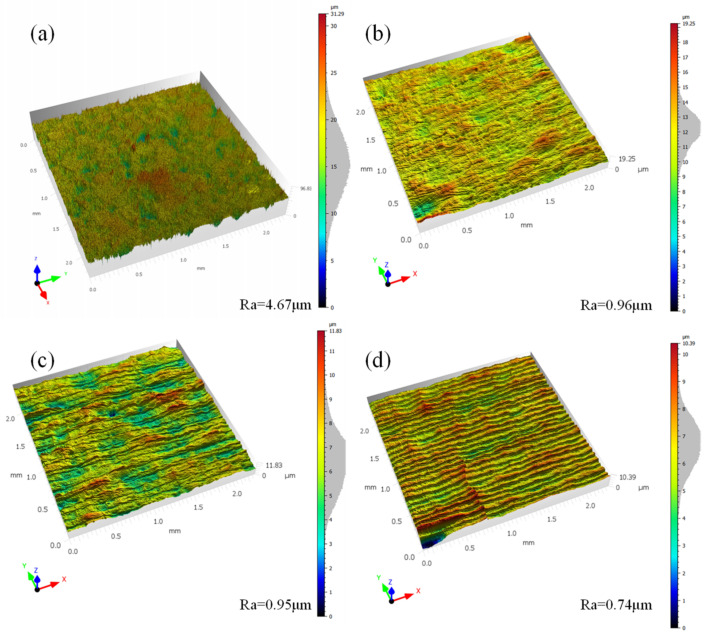
Surface morphology of alumina before and after polishing. (**a**) 0#, original surface, (**b**) 1#, normal temperature, (**c**) 2#, preheating 450 °C, (**d**) 3#, preheating 900 °C.

In order to obtain the best combination of the polishing process parameters (laser power, scanning speed, pulse frequency, and scan spacing set in the original parameters) and the optimization of the target for the minimum value of surface roughness, we used the software to obtain the optimization results, namely, a scanning speed of 323.5 mm/s, a laser power of 73.63 W, a pulse frequency of 2.3 kHz, and a scan spacing of 0.09 mm. With the combination of the obtained laser polishing process parameters from the laser polishing experiments, we used laser confocal microscopy to measure the surface roughness. Figure 5 shows the sample polishing before and after the surface morphology comparison. Figure 5a shows the original surface morphology; it can be seen that the surface undulation is more obvious, mainly due to the sintering process, which is not uniform in the distribution of surface powder particles and has a surface roughness of 4.67 μm. Figure 5b shows the response surface optimization. Figure 5b shows the polished surface morphology obtained by using the response surface to optimize the optimal process parameters; it can be seen from the figure that the surface undulation is obviously smaller after polishing; the surface roughness is reduced to 0.96, and the reduction rate of surface roughness is 79.4%; the surface roughness is further reduced compared with the reduction rate of 78% before optimization.

### 3.2. Changes in Material Surface Properties after Flame Preheating and Polishing

**Figure 6:**
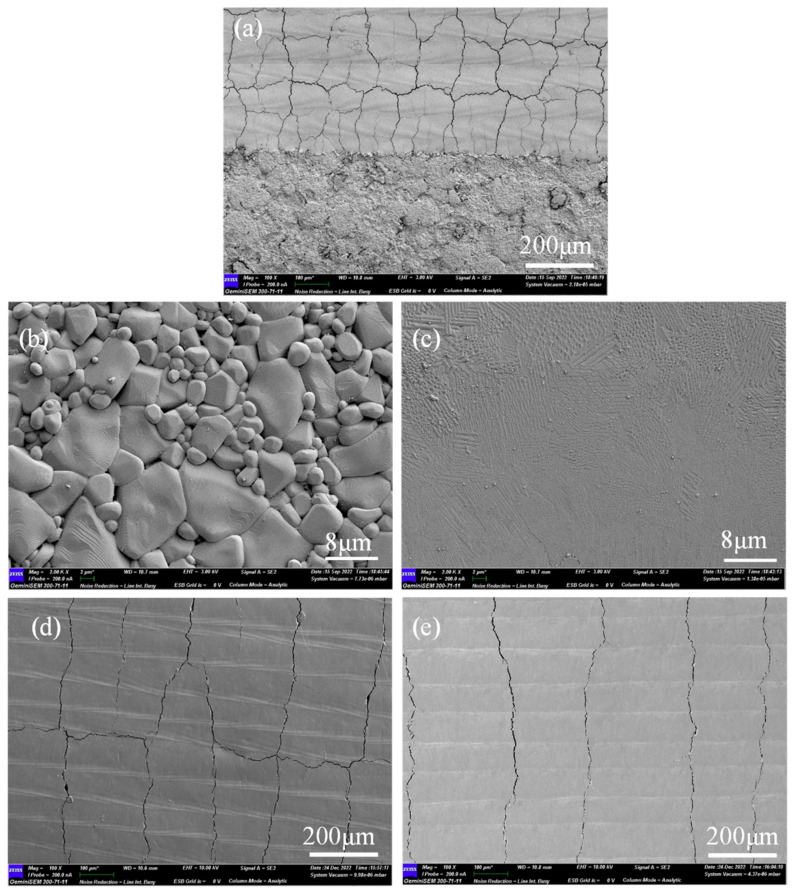
SEM images of the surface before and after laser polishing. (**a**) 1#, polished at normal temperature, (**b**) local magnification of the original surface of alumina, (**c**) local magnification of the polished surface at normal temperature, (**d**) 2#, preheated at 450 °C, (**e**) 3#, preheated at 900 °C.

In order to minimize the crack defects on the surface of the material and improve the surface quality, the flame-assisted laser polishing of alumina ceramics was used to increase the surface temperature of the material through flame preheating and reduce the residual thermal stress generated by the temperature difference during laser polishing, thus reducing the generation of surface cracks. Figure 5 shows the surface morphology of alumina ceramics before and after polishing, and the processing laser process parameters are the best process parameters obtained from response surface optimization. Figure 5a shows the surface morphology of unpolished alumina ceramics. Figure 5b–d show the surface morphology at normal temperature, preheating at 450 °C, and preheating at 900 °C. From the figure, it can be seen that the surface roughness of the material decreases significantly after laser polishing and by preheating the material. After the treatment, the surface roughness is further reduced to 0.74 μm. Figure 6 shows the SEM image of the alumina ceramic surface before and after laser polishing. From Figure 6a, a large number of microcracks can be found on the material surface after laser scanning compared to the unpolished surface. In Figure 6b, we can clearly see the ceramic surface after sintering large particles of powder and voids. The polished surface is melted and flowing due to the laser radiation, so the surface of the material is effectively smoothed and the surface is free of large particles and voids. Among them, Figure 6a shows the surface obtained from polishing at normal temperature, and Figure 6d,e show the surfaces obtained from polishing at temperatures of 450 and 900 °C. Comparing the distribution of cracks on the surface of the material at different temperatures, it can be found that the surface cracks of the material have improved significantly after the preheating treatment, especially the longitudinal cracks on the surface. At 900 °C, the longitudinal cracks on the polished surface are observed to have basically disappeared. According to previous studies, it was found that when the laser spot scanned the material surface, the material at the spot began to melt, while the material at the previous spot scan began to solidify and shrink. The surface material melting, solidification, and shrinkage process is not consistent, so the processing surface accumulates a large amount of thermal stress. At the same time, due to the short liquid phase time, the entire sample structure is loose and has a low tensile limit. When the internal stress caused by the laser exceeds the material tensile limit, the material surface releases the stress by generating transverse cracks perpendicular to the scanning direction [19]. The source of longitudinal cracks is mainly solidification, and during laser polishing, the surface material re-solidifies from the boundary to the center of the melt pool while the material solidifies and shrinks from the center of the melt pool to the boundary. It is this shrinkage that generates tensile stresses toward the boundary and extends along the center of the track to produce longitudinal cracks [20]. When the material is preheated, the temperature difference between the melt pool during processing decreases and the solidification rate of the melt pool decreases, so there are fewer longitudinal cracks on the polished surface compared to the non-preheated polished samples. When the material was preheated to 1600 °C, the surface cracks disappeared completely after laser polishing [21]. Combined with Figure 5, it can be speculated that the reduction in surface roughness may be caused by the reduction in cracks.

Residual stress is an important factor affecting the surface quality of polished alumina ceramics by laser. Table 5 gives the residual stress on the original surface of alumina ceramics and the polished surface at different preheating temperatures; the model with the LXRD high-speed residual stress tester was used in the experiments.

Combined with Table 5 and Figure 6, it can be seen that preheating before polishing can significantly reduce the surface crack density, mainly because the preheating temperature reduces the temperature difference in the cooling process so that there is less cooling shrinkage of the remelted layer after polishing. Table 5 shows that the size of the polished surface residual stress after preheating is significantly lower than the surface residual stress of direct laser polishing at a normal temperature. When the preheating temperature reaches 900 °C, the surface residual stress reaches 100.8 ± 20.0 MPa; the surface residual stress of laser polishing at a normal temperature directly decreased by more than 50%. These results again prove that preheating before laser polishing can significantly reduce the polishing time. These results also demonstrate the fact that preheating before laser polishing can significantly reduce the thermal stresses generated during polishing.

**Figure 7:**
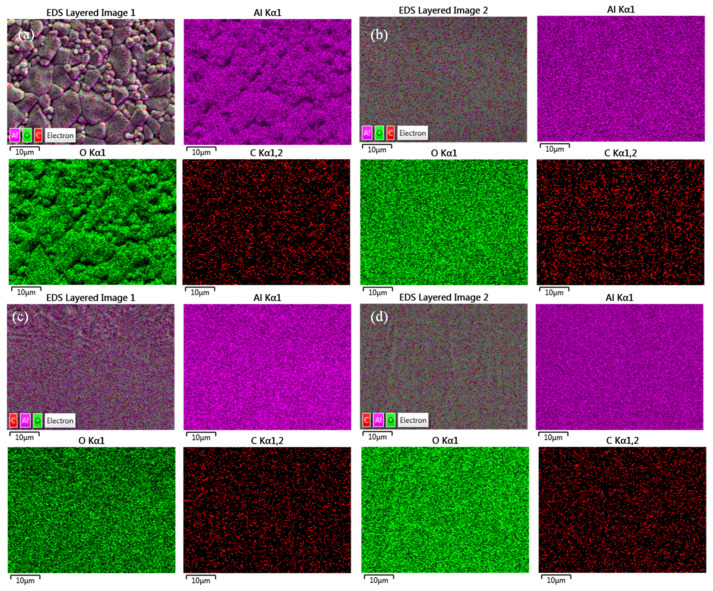
Elemental analysis before and after EDS polishing. (**a**) 0#, original surface, (**b**) 1#, normal temperature, (**c**) 2#, preheating 450 °C, (**d**) 3#, preheating 900 °C.

**Figure 8:**
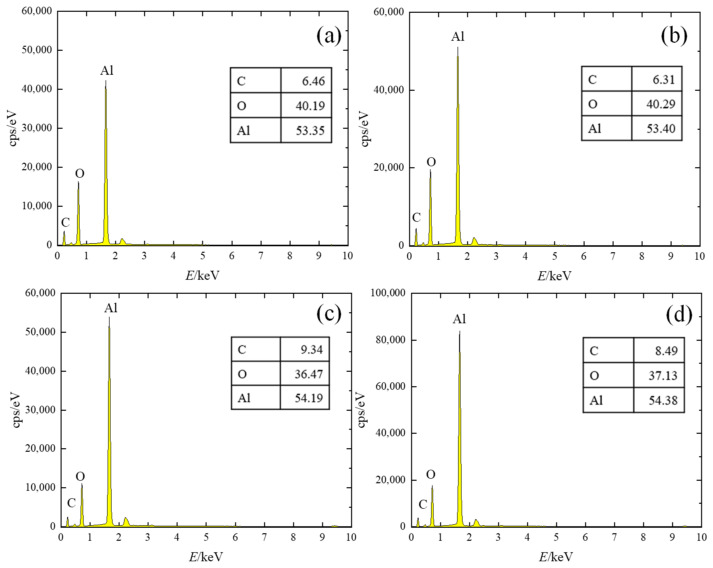
Distribution of elemental content of alumina surface before and after polishing. (**a**) 0#, original surface, (**b**) 1#, normal temperature, (**c**) 2#, preheating 450 °C, (**d**) 3#, preheating 900 °C.

**Figure 9:**
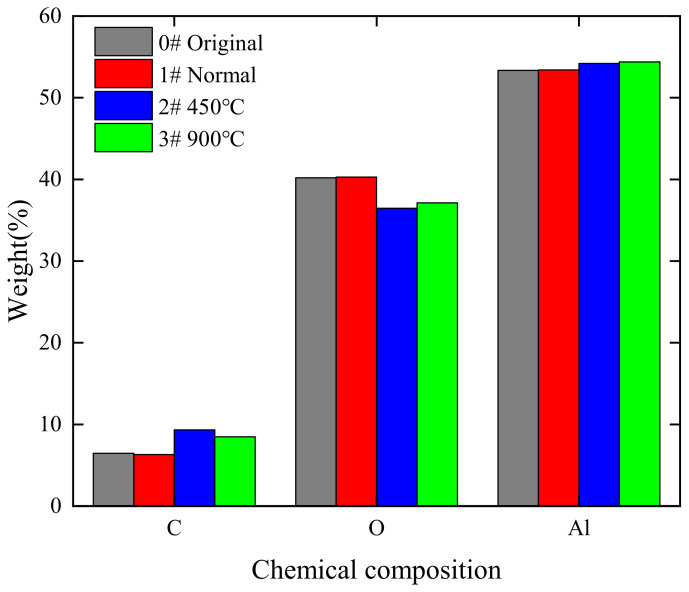
Distribution of elemental content under different processing conditions.

In order to analyze the change of material surface elements before and after polishing, an EDS surface sweep was performed on the original surface and the polished surface. It can be seen in Figure 7 that the material surface elements are evenly distributed before and after processing, and the laser polishing process does not affect the material surface element distribution. Figure 8 shows the surface element content of the material before and after pre-polishing; from the figure, it can be seen that the main elements of the material surface are C, O, and Al, while the element content of C may be due to experimental operation pollution, which cannot be completely avoided. Figure 9 shows the histogram of surface element distribution under different processing conditions. Comparing the elemental content of the surface before and after polishing, it can be found that there is almost no change in the elemental content of the surface, which may be mainly due to the fact that no chemical reaction occurs in high-purity alumina ceramics during laser polishing; only the solid–liquid phase change of the material occurs during the polishing process.

**Figure 10:**
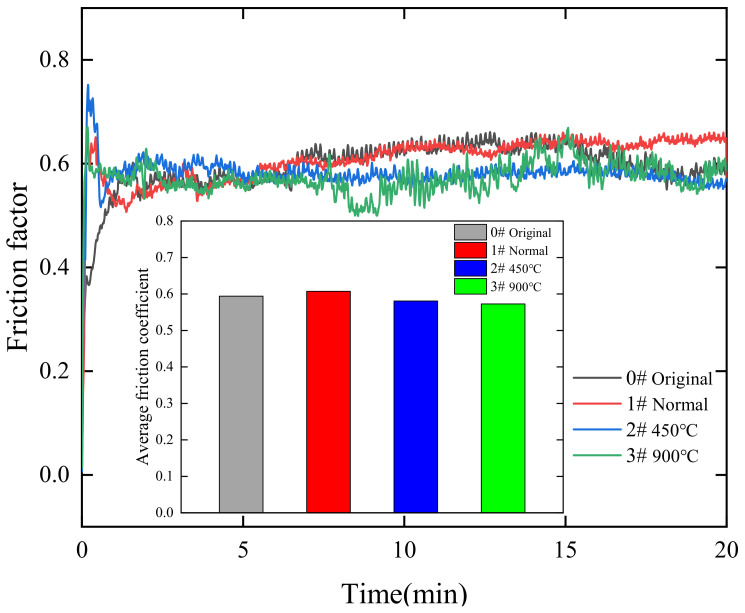
Changes in surface friction properties before and after laser polishing.

**Figure 11:**
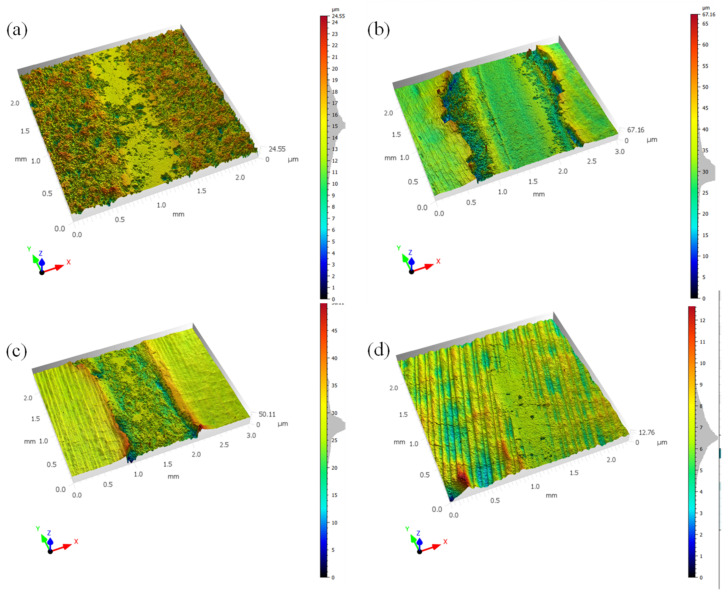
Friction wear track morphology. (**a**) 0#, original surface, (**b**) 1#, normal temperature, (**c**) 2#, preheating 450 °C, (**d**) 3#, preheating 900 °C.

In order to study the changes in the surface properties of alumina ceramics after laser polishing, friction and wear tests were conducted on the polished surfaces at the original normal temperature, 450 °C, and 900 °C. This experiment was conducted by dry friction, and the material of the grinding ball was Si_3_N_4_. The test was conducted with a load of 10 N and a motor speed of 300 rpm for 20 min. Figure 10 shows the change in surface friction properties after laser polishing at different temperatures, from which it can be concluded that the average friction coefficient of the original surface of the alumina ceramic is 0.5936, and the average friction coefficient of the polished surface at normal temperature is 0.6074; the average friction coefficient of the surface of the material gradually decreases after polishing by preheating, and when the preheating temperature is 900 °C, the average friction coefficient of the surface is 0.5725 lower than that of the original surface and the average friction coefficient of the laser-polished surface at normal temperatures. From the change in the surface average friction coefficient, it can be seen that the wear resistance of laser-polished alumina ceramics has been improved after preheating, and within a certain range, the preheating temperature is conducive to the improvement of surface wear resistance. In order to study the change in surface friction properties more intuitively, Figure 11 shows the surface friction wear track morphology of the polished surface. When comparing the surface wear track width before and after polishing, it can be found that the normal temperature and 450 °C temperature laser polishing surface wear track widths, relative to the original surface width, increase. When the preheating temperature increases to 900 °C, the polished surface wear track width gradually becomes narrower. The surface wear track width change may be due to the preheating caused by the surface crack reduction; at the same time, the crack generation will make the grinding ball’s scraping effect on the material surface increase. Alumina ceramics are brittle materials, so the surface crack generation will make the friction track width increase; after the laser polishing, the surface roughness is reduced; however, the surface wear resistance increases through the material preheating treatment. By preheating the material, the surface cracks are reduced, the wear track is gradually narrowed, and the surface wear resistance of the material increases compared to the original surface and surfaces polished at normal temperatures.

**Figure 12:**
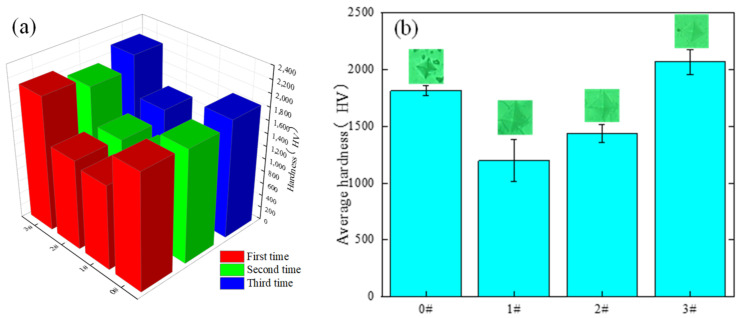
Laser polishing surface hardness surface change. (**a**) change in surface hardness of alumina, (**b**) change in average surface hardness.

Figure 12 shows the change in the surface hardness of the material after laser polishing. The experiment uses a Vickers hardness tester; the load used in the test is 0.3 kg. In order to ensure the accuracy of the test results, each group of experimental data was tested three times. Taking the average value, from the test results, it can be seen that the average hardness of the original surface of the material is 1810 HV, and the surface hardness after laser polishing at a normal temperature drops to 1197 HV. With the preheating treatment of the material, after the preheating process, the laser polishing of the alumina surface gradually increases the hardness. When the preheating temperature reaches 900 °C, the laser polishing of the material increases the surface hardness to 2063 HV; compared to the original surface hardness, it is increased by 13.9%. For the experimental decrease in material surface hardness after laser polishing at a normal temperature, it may be due to the numerous cracks generated by laser polishing at a normal temperature, leading to surface material sparseness, which can be clearly seen from the indentation in Figure 12b. The directly polished surface indentation without the preheating process easily produces cracks, and the polished surface cracks are suppressed after the preheating process treatment. The material surface remelting due to laser polishing produces, at the same time, an increase in the surface hardness of laser-polished alumina ceramics due to the dense layer produced by the remelting of the material surface caused by laser polishing.

**Figure 13:**
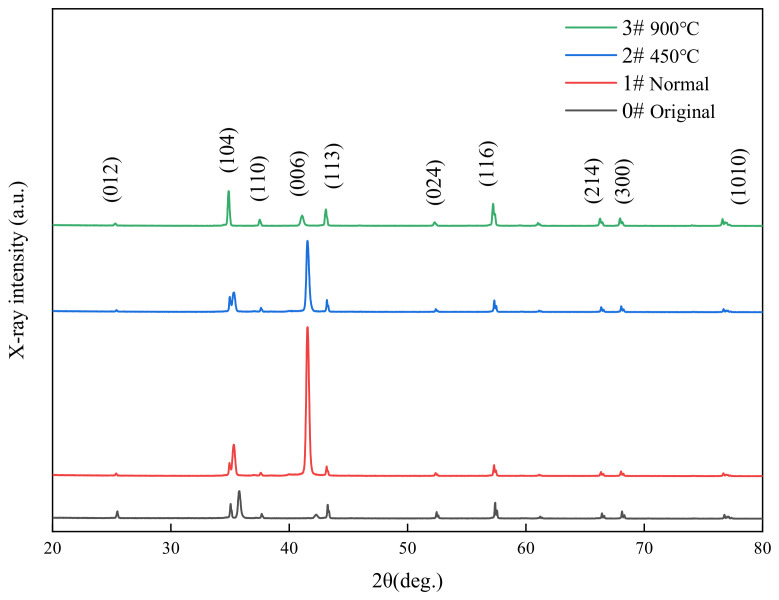
XRD spectra of laser-polished alumina ceramics before and after polishing.

The XRD pattern analysis of the sample is shown in Figure 13, with a detection angle of 20–80 degrees. As the sample used was a high-purity alumina ceramic, the sample was made using a conventional sintering process, and it is known that α-Al_2_O_3_ is the most stable alumina phase at temperatures above 1200 °C; other sub-stable (e.g., γ, δ, θ, and η) alumina is known as transition alumina [22] and, therefore, is considered as the main reason for the absence of a phase change in sintering and laser processing. The main α-Al_2_O_3_ peaks were labeled according to the reference XRD pattern (ICDDPD card No. 46-1212), and the change in grain size before and after polishing was analyzed qualitatively in experiments by the full width at the half-peak (FWHM) size. In general, the smaller the grain, the more severe the broadening, and vice versa. The FWHM of the selected (006) crystalline surface was 0.238 before polishing, while the actual FWHMs were 0.260 and 0.261 after polishing at normal temperature and 460 °C preheating, respectively, and 0.254 after polishing at 900 °C preheating, so it can be seen that the grain size is somewhat refined before and after polishing, which is also consistent with the morphological changes before and after polishing in the previous SEM micrographs.

Based on the above experimental data, it can be concluded that the surface roughness of alumina ceramics can be effectively reduced by using CO_2_ laser polishing, which is consistent with the results of Bharatish et al. [10]. The surface residual stress was greatly reduced by laser polishing after preheating, which is consistent with the results of Das et al. [18]. In addition, the SEM, XRD, hardness, and friction wear tests revealed that laser polishing can effectively refine the powder particles on the material surface, and preheating polishing can increase the surface hardness and wear resistance.

## 4. Conclusions

1. The response surface test was used to optimize the laser process parameters, and the best laser polishing process parameters were finally determined as a scanning speed of 323.5 mm/s, a laser power of 73.63 W, a pulse frequency of 2.3 kHz, and a scanning spacing of 0.09 mm; the surface roughness of the material could be reduced from 4.67 to 0.96 μm using the combination of the process parameters, and the surface roughness was reduced by 79.4%.
2. Using the preheating process to reduce the cooling rate of laser-polished alumina ceramic surfaces, the polished surface microcracks were suppressed, and the surface roughness of the material was reduced to 0.74 μm after laser polishing at 900 °C; the surface roughness was reduced by 84.2%.
3. The surface before and after polishing was tested by SEM, EDS, XRD, residual stress, hardness, friction wear, and other testing methods. The results showed that laser polishing can effectively refine the powder particles on the surface of alumina, and the residual stress on the surface decreased by about 53.6% after laser polishing after preheating at 900 °C. Meanwhile, the surface hardness and wear resistance were improved.

## Figures and Tables

**Table 1:** The 99 alumina ceramic compositions.

Al_2_O_3_	SiO_2_	Fe_2_O_3_	Na_2_O	Others
≥99.00	≤0.10	≤0.10	≤0.40	≤0.40

**Table 2:** Response surface experimental factor level table.

Level	Factors			
	Scanning Speed *v* (mm/s)	Laser Power *P* (W)	Pulse Frequency *f* (kHz)	Scan Spacing *b* (mm)
−1	120	40	1	0.04
0	320	70	2.5	0.08
1	520	100	4	0.12

**Table 3:** Response surface experimental design and surface roughness.

No.	*v* (mm/s)	*P* (W)	*f* (kHz)	*b* (mm)	*R*a (μm)
1	120	40	2.5	0.08	1.812
2	520	40	2.5	0.08	2.17
3	120	100	2.5	0.08	2.05
4	520	100	2.5	0.08	1.57
5	320	70	1	0.04	1.662
6	320	70	4	0.04	3.02
7	320	70	1	0.12	1.98
8	320	70	4	0.12	1.547
9	120	70	2.5	0.04	2.92
10	520	70	2.5	0.04	1.67
11	120	70	2.5	0.12	1.32
12	520	70	2.5	0.12	1.93
13	320	40	1	0.08	1.52
14	320	100	1	0.08	1.89
15	320	40	4	0.08	2.64
16	320	100	4	0.08	1.75
17	120	70	1	0.08	1.411
18	520	70	1	0.08	1.38
19	120	70	4	0.08	1.89
20	520	70	4	0.08	1.53
21	320	40	2.5	0.04	2.31
22	320	100	2.5	0.04	2.98
23	320	40	2.5	0.12	2.45
24	320	100	2.5	0.12	1.56
25	320	70	2.5	0.08	1.013
26	320	70	2.5	0.08	1.081
27	320	70	2.5	0.08	1.192
28	320	70	2.5	0.08	1.235
29	320	70	2.5	0.08	1.004

**Table 4:** Response surface ANOVA results.

Source	Sum of Squares	Mean Square	*F*_value_	*P*_value_	Significance
Model	9.05	0.65	77.71	<0.0001	significant
A-*v*	0.11	0.11	13.32	0.0026	
B-P	0.1	0.1	12.17	0.0036	
C-*f*	0.54	0.54	64.32	<0.0001	
D-*b*	1.19	1.19	142.75	<0.0001	
AB	0.18	0.18	21.1	0.0004	
AC	0.027	0.027	3.25	0.0929	
AD	0.86	0.86	103.97	<0.0001	
BC	0.4	0.4	47.71	<0.0001	
BD	0.61	0.61	73.14	<0.0001	
CD	0.8	0.8	96.4	<0.0001	
Residual	0.12	8.32 × 10^−3^			
Lack of Fit	0.073	7.28 × 10^−3^	0.67	0.7263	not significant
Pure Error	0.044	0.011			
Cor Total	9.17				
R^2^				0.9873	
R^2^_Adj_				0.9746	

**Table 5:** Residual stresses on laser-polished alumina surfaces at different preheating temperatures.

Materials	Laser Polishing Parameters	Preheating Temperature (°C)	Residual Stress (MPa)
99 Alumina Ceramic	power 73.63 W, frequency 2.3 kHz, scanning speed 323.5 mm/s, scanning spacing of 0.09 mm	Normal	217.3 ± 15.9
		450	125.8 ± 21.2
		900	100.8 ± 20.0

## Data Availability

The data presented in this study are available on request from the corresponding author.
